# Review of genus *Pseudorthocladius* Goetghebuer, 1943 (Diptera, Chironomidae) from China

**DOI:** 10.3897/zookeys.387.5808

**Published:** 2014-03-11

**Authors:** Jing Ren, Xiaolong Lin, Xinhua Wang

**Affiliations:** 1 College of Life Sciences, Nankai University, Tianjin 300071, China

**Keywords:** Chironomidae, *Pseudorthocladius*, new species, new record, key, Oriental, China

## Abstract

The genus *Pseudorthocladius* Goetghebuer, 1943 from China, including 12 species, is reviewed. Five new species, *P. (P.) binarius* **sp. n.**, *P. (P.) cylindratus* **sp. n.**, *P. (P.) digitus* **sp. n.**, *P. (P.) ovatus* **sp. n.** and *P. (P.) paucus* **sp. n.** are described and illustrated as adult males. *P. (P.) cristagus* Stur & Sæther, 2004, *P. (P.) jintutridecima* (Sasa, 1996), *P. (P.) macrovirgatus* Sæther & Sublette, 1983, *P. (P.) morsei* Sæther & Sublette, 1983, *P. (P.) uniserratus* Sæther & Sublette, 1983, *P. (L.) wingoi* Sæther & Sublette, 1983 are newly recorded in Oriental Region. A key to the males of *Pseudorthocladius* in China is presented.

## Introduction

The genus *Pseudorthocladius* Goetghebuer, 1943 contains two subgenera, *Pseudorthocladius* Goetghebuer and *Lordella* Sæther & Sublette (Sæther and Sublette 1983). The two subgenera are different in the shape of inferior vosella and microtrichia on the gonostylus. The presence of well developed pulvilli [except *Pseudorthocladius (Pseudorthocladius) oyabecrassus* Sasa, Kawai & Ueno, 1988], an apical antennal seta, lack of pseudospurs, acrostrichals long and beginning near the antepronorum, curved Cu_1_ and an anal point with strong setae will separate the genus from other orthoclad genera.

The subgenus *Lordella* shows some similarities and possible synapomorphies with *Doithrix*, such as the basally widened gonostylus and hook–shaped inferior volsella. However, the long anal point and setae on the point indicating an intermediate position between typical *Pseudorthocladius* s. str. and *Doithrix*. So the status of subgenus *Lordella* needs to be further discussed.

The immatures of *Pseudorthocladius* are found in a wide variety of damp habitats including mosses, hygropetric regions, seeps and floodplains along stream banks (Strenzke 1950, Sæther and Sublette 1983, Cranston et al. 1989)

According to Ashe and O’Connor 2012, this genus presently comprises 52 valid species in the world with 34 species in the Palaearctic Region, 17 in the Nearctic Region, 5 in the Oriental Region, 2 in the Afrotropical Region. Eastern Palaearctic Asia appears to be a rich area of diversity in the genus: 24 species from Japan (Yamamoto 2004), 7 species from the Far East of Russia (Makarchenko and Makarchenko 2012), only 1 species from China (Wang 2000).

In this paper, the *Pseudorthocladius* based on material from China is reviewed. Five new species are described, six species are newly recorded in China, and key to the Chinese species of *Pseudorthocladius* is presented.

## Materials and methods

The morphological nomenclature follows Sæther (1980). The materials examined are mounted on slides, following the procedure outlined by Sæther (1969). Measurements are given as ranges followed by the mean, when four or more specimens are measured, followed by the number of specimens measured (n) in parentheses. All specimens examined during this study are deposited in the College of Life Sciences, Nankai University, China.

## Taxonomy

### Pseudorthocladius (Pseudorthocladius) binarius sp. n.

http://zoobank.org/D5AC53EC-771A-4D91-ADF2-B3EB97CDF0FD

http://species-id.net/wiki/Pseudorthocladius_binarius

Figures 1–7

#### Diagnosis.

The male imago can be distinguished from the known species of the genus by the following combination of characters: low AR (0.29); squama with few setae; anal lobe reduced; inferior volsella has two sub–lobes; virga absent.

#### Description.

Adult male (n = 4). Total length 1.50–1.80, 1.63 mm. Wing length 0.81–0.97, 0.89 mm. Total length/wing length 1.83–1.86, 1.84. Wing length/length of profemur 2.26–2.43 (3).

Coloration. Head, abdomen, legs brown; thorax with yellow ground with brown postnotum and preepisternum.

Head. Antenna with 13 flagellomeres. Terminal flagellomere length 95–108, 105 μm. AR 0.28–0.33, 0.29. Temporal setae 8 (2), including 2 (2) inner verticals, 3–4 (3) outer verticals, and 2 (2) postorbitals. Clypeus with 6–10, 8 setae. Tentorium 86–96, 92 μm long, 14–19, 15 μm wide. Palpomere lengths (in μm): 19–24, 22; 26–29, 28; 55–62, 59; 84–91, 86; 98–120, 110. L: 5^th^/3^rd^ 1.77–2.10, 1.86.

**Figures 1–7.**
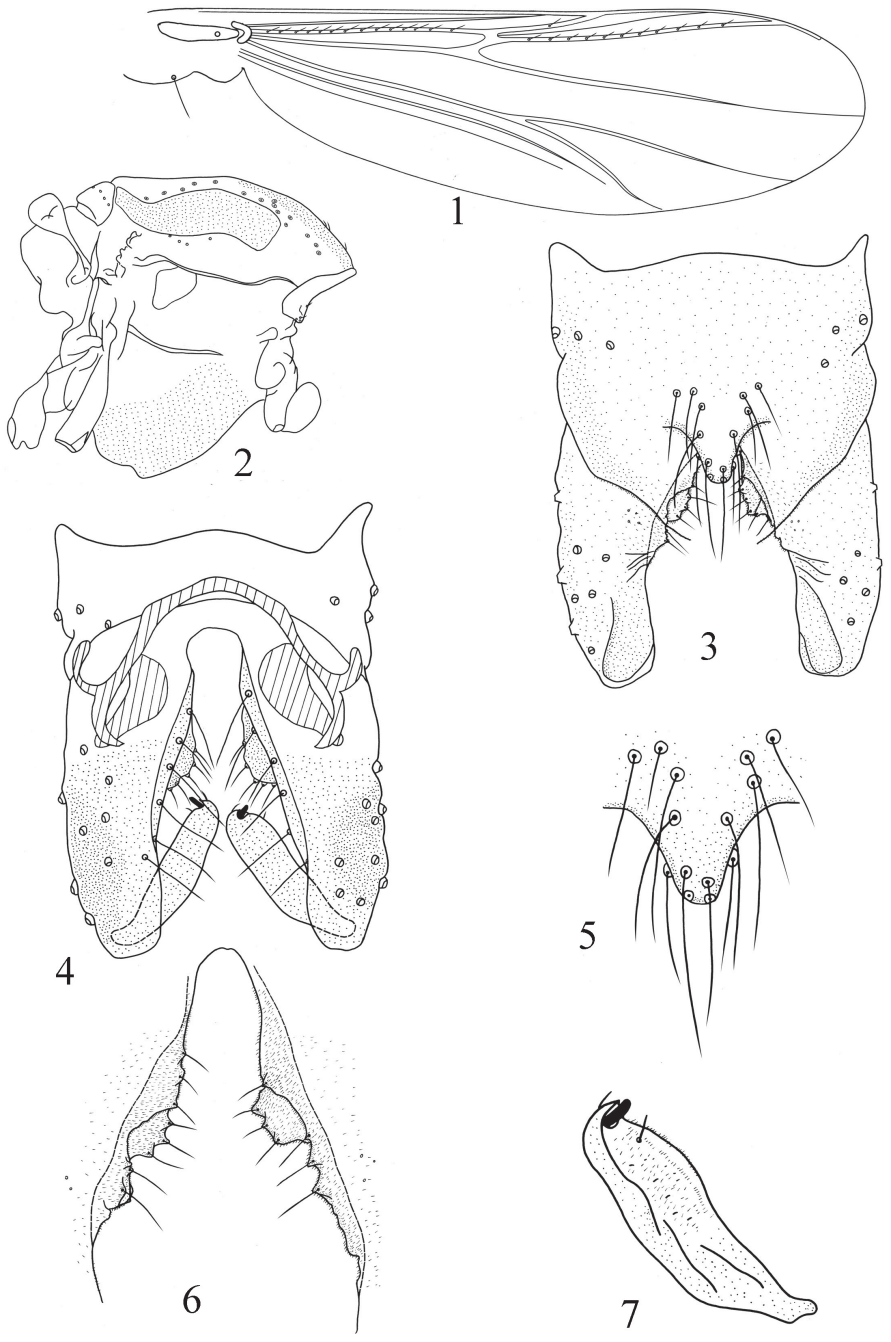
*Pseudorthocladius (Pseudorthocladius) binarius* sp. n., male. **1** wing **2** thorax **3** hypopygium (dorsal view) **4** hypopygium (ventral view) **5** anal point **6** inferior volsella **7** gonostylus.

Wing (Figure 1). VR 1.25–1.29 (3). Anal lobe reduced. Brachiolum with 1 seta; R with 9–15, 13 setae; R_1_ with 1–4, 2 setae; R_4+5_ 9–11, 10 setae; M with 0–1, 1 seta. Squama with 1–2, 1 seta. Costal extension 70 μm long. Cu_1_ slightly curved.

Thorax (Figure 2). Antepronotum with 3–6, 5 lateral setae, dorsocentrals 13–15, 13, acrostichals 3–7, 5, prealars 4–6, 5. Scutellum with 6–8, 7 setae.

Legs. Pulvilli present. Spur of fore tibia 29 (3) μm long, spurs of mid tibia 22–24, 23 μm and 16–19, 18 μm long; hind tibia with a long spur 40–43, 42 μm long, a short spur 14–17, 16 μm long and comb composed of 10–12, 11 spines. Width at apex of fore tibia 24–28, 26 μm, of mid tibia 20–26, 24 μm, of hind tibia 31–36, 33 μm. Lengths (in μm) and proportions of legs as in Table 1.

**Table 1.** Lengths (in μm) and proportions of legs of *Pseudorthocladius (Pseudorthocladius) binarius* sp. n.

	p_1_	p_2_	p_3_
fe	350–400 (3)	350–400, 375	360–400, 380
ti	340–420 (3)	350–430, 373	410–500, 435
ta_1_	250–300 (2)	125–150, 131	220–260, 240
ta_2_	170–210 (2)	72–96, 78	115–130, 124
ta_3_	120–140 (2)	60–72, 63	105–126, 115
ta_4_	72–84 (2)	36–48, 42	48–60, 53
ta_5_	51–67 (2)	48–50, 49	48–60, 54
LR	0.71 (2)	0.33–0.37, 0.35	0.52–0.56, 0.55
BV	2.22–2.30, 2.29	3.63–3.98, 3.71	2.98–3.13, 3.02
SV	2.73–2.80, 2.76	5.53–5.72, 5.62	3.32–3.50, 3.41
BR	2.50–2.60, 2.56	3.14–3.67, 3.45	3.75–4.38, 4.03

Hypopygium (Figures 3–7). Laterosternite IX with 4–6, 5 setae. Anal point (Figure 5) subtriangular with rounded apex, 24–26, 25 μm long, with 13–15, 14 strong setae. Phallapodeme 22–24, 24 μm long. Transverse sternapodeme 44–50, 48 μm long. Virga absent. Gonocoxite 110–122, 118 μm long. Inferior volsella (Figure 6) with two sub–lobes, the dorsal lobe with concave inner margin and 4–5, 5 marginal setae, the ventral lobe semi–rounded with 3–4, 3 marginal setae. Gonostylus (Figure 7) 60–67, 64 μm long, narrowed at base. Megaseta 9–10, 10 μm long. HR 1.82–1.92, 1.85. HV 2.43–2.48, 2.45.

Female, pupa and larva unknown.

#### Type materials.

Holotype: ♂ (BDN No.20200), China, Fujian, Quanzhou City, Dehua County, Daiyun Mountain, 25°40'N, 118°11'E, 13.ix.2002, Zheng Liu, sweep net. Paratypes: 3 ♂♂, as holotype.

#### Etymology.

The specific name is from Latin, *binarius*, meaning “of two”, referring to the inferior volsella has two sub–lobes.

#### Remarks.

The new species resembles *Pseudorthocladius (Pseudorthocladius) tusimoquereus* Sasa & Suzuki (1999) in the structure of hypopygium, but can be separated from the latter on the following points: (1) *Pseudorthocladius (Pseudorthocladius) binarius* sp. n. has small body size (1.63 mm) and low AR (0.29); (2) wing anal lobe reduced and squama with few setae; (3) inferior volsella with two sub–lobes; (4) virga absent.

#### Distribution.

The new species is collected in a subtropical mountain area in Fujian Province (Oriental China).

### Pseudorthocladius (Pseudorthocladius) cristagus

Stur & Sæther, 2004

http://species-id.net/wiki/Pseudorthocladius_cristagus

Pseudorthocladius (Pseudorthocladius) cristagus Stur & Sæther, 2004: 79; Ashe and O’Connor 2012: 531.

#### Diagnosis.

The male imago is separable from the other species of the genus *Pseudorthocladius* by having hairy wings, strong crista dorsalis and outer heel of the gonotylus.

#### Specimens examined.

China, Zhejiang: 1 ♂ (BDN No.K5B50), Taizhou City, Tiantai County, Huading Mountain, 29°15'45"N, 121°06'36"E, 13.iv.2011, Xiaolong Lin, sweep net.

#### Remarks.

Stur and Sæther (2004) erected a hairy–winged species *Pseudorthocladius (Pseudorthocladius) cristagus* based on the specimen from Luxemburg. The species can be separated from close species *Pseudorthocladius (Pseudorthocladius) pilosipennis* by having a gonostylus with a prominent crista dorsalis and an outer heel. The Chinese specimen mainly agrees with the original description of Stur and Sæther (2004). Some measured differences between the specimens from China and Luxemburg are shown in Table 2.

**Table 2.** Differences between the specimens from China and Luxemburg.

	Chinese specimen	Luxemburg specimens
TL	2.48 mm	3.35–3.41 mm
WL	1.63 mm	1.89–2.02 mm
AR	1.05	1.08–1.14
LR_1_	0.62	0.75–0.76

#### Distribution.

Zhejiang Province (Oriental China); Luxemburg.

### Pseudorthocladius (Pseudorthocladius) curtistylus

(Goetghebuer, 1921)

http://species-id.net/wiki/Pseudorthocladius_curtistylus

Hydrobaenus (Psectrocladius) curtistylus Goetghebuer, 1921: 101.
Spaniotoma (Orthocladius) curtistylus (Goetghebuer); Edwards 1929: 350.
Orthocladius (Pseudorthocladius) curtistylus (Goetghebuer); Goetghebuer 1932: 93, 1940–50: 73.
Spaniotoma curtistylus (Goetghebuer); Edwards 1932: 141.
Hydrobaenus (Pseudokiefferiella) curtistylus (Goetghebuer); Laurence 1951: 165.
Pseudorthocladius curtistylus (Goetghebuer); Thienemann and Kruger 1939: 25; Thienemann 1944: 569, 616; Coe 1950: 160; Strenzke 1950: 230; Brundin 1956: 137; Lehmann 1971: 459; Pinder 1978: 94.
Pseudorthocladius (Pseudorthocladius) curtistylus (Goetghebuer): Sæther and Sublette 1983: 69, fig. 37; Wang 2000: 639; Ashe and O’Connor 2012: 532; Makarchenko and Makarchenko 2012: 76–77.

#### Diagnosis.

AR 0.45–0.70; dorsocentrals 15–18; R with 9–13 setae, R_1_ with 2–3 setae, R_4+5_ with 0–14 setae; squama with 3–4 setae; virga present. Type I with low and extending crista dorsalis, type II with crista dorsalis absent, type III with round and protruding crista dorsalis.

#### Specimens examined.

China, Zhejiang: 3 ♂♂, Wenzhou City, Taishun County, 27°33'N, 119°39'E, 1.viii.2005, Xin Qi, light trap; Tianmu Mountain, 30°19'N, 119°26'E, 23.vi.1998, Bingchun Ji, sweep net. Fujian: 10 ♂♂, Wuyi City, Wuyi Mountain, 27°45'N, 118°03'E, 30.iv.1993, Xinhua Wang, sweep net. Guangdong: 5 ♂♂, Fengkai County, 23°24'N, 111°30'E, 20.iv.1988, Xinhua Wang, sweep net. Yunnan: 2 ♂♂, Dali Bai Autonomous Prefecture, Cang Mountain, Qingbi River, 25°36'N, 100°15'E, 23.v.1996, Yuzhou Du, light trap. Hunan: 2 ♂♂, Zhuzhou City, Yanling County, 26°27'N, 113°42'E, 16.vii.2004, Chuncai Yan, sweep net.

#### Remarks.

The Chinese specimens agree with the description of *Pseudorthocladius (Pseudorthocladius) curtistylus* type II and type III. According to Sæther and Sublette (1983), type II without crista dorsalis, while type III with rounded and protruding crista dorsalis.

#### Distribution.

The species is widely distributed in Holarctic region.

### Pseudorthocladius (Pseudorthocladius) cylindratus sp. n.

http://zoobank.org/399C86DD-F23B-4C2B-9E9F-E8711959DCA2

http://species-id.net/wiki/Pseudorthocladius_cylindratus

Figures 8–13

#### Diagnosis.

The male imago can be distinguished from the known species of the genus by the following combination of characters: cylindrical anal point; well–developed triangular inferior volsella; low AR (0.66) and high VR (1.37).

#### Description.

Adult male (n = 6). Total length 1.68–1.97, 1.87 mm. Wing length 1.04–1.19, 1.15 mm. Total length/wing length 1.53–1.75, 1.63. Wing length/length of profemur 2.64–2.88, 2.71.

Coloration. Head, abdomen, legs light brown; thorax with light brown ground with brownish black postnotum and preepisternum.

Head. Antenna with 13 flagellomeres. Terminal flagellomere length 235–264, 254 μm. AR 0.65–0.67, 0.66. Temporal setae 8–10, 9, including 3–6, 4 inner verticals, 4–5, 5 outer verticals, and 0–2, 1 postorbital. Clypeus with 8–11, 10 setae. Tentorium 96–113, 103 μm long, 18–21, 19 μm wide. Palpomere lengths (in μm): 22–26, 24; 24–28, 26; 60–72, 65; 84–91, 86; 113–137, 130. L: 5^th^/3^rd^ 1.87–2.28, 2.00.

**Figures 8–13.**
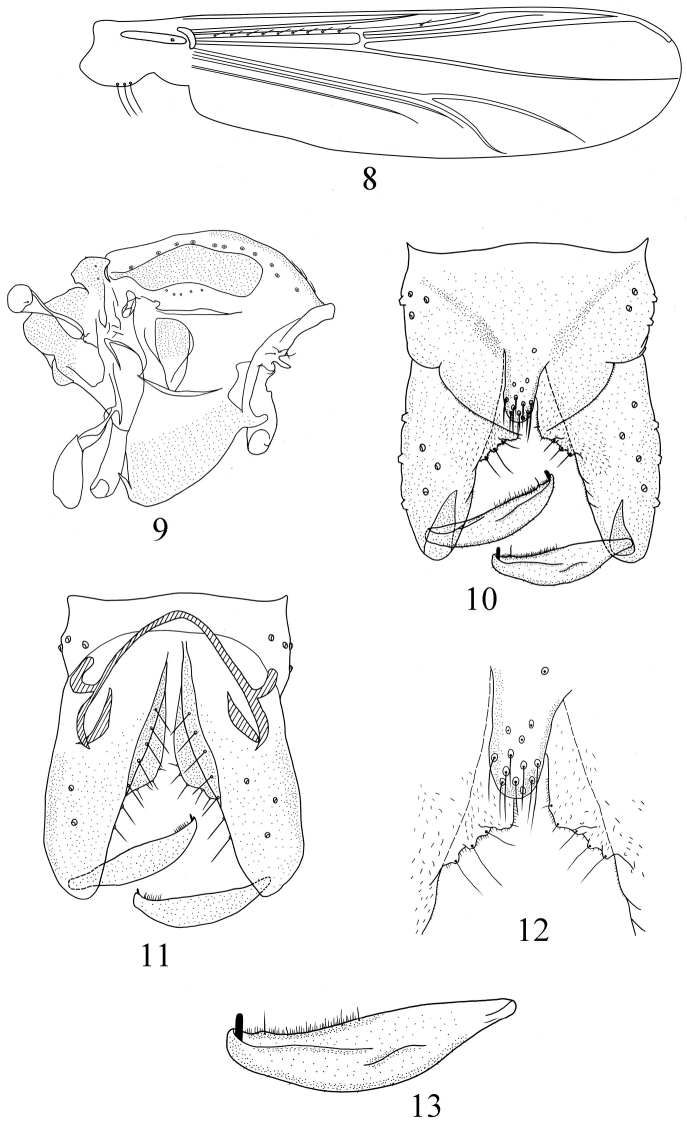
*Pseudorthocladius (Pseudorthocladius) cylindratus* sp. n., male. **8** wing **9** thorax **10** hypopygium (dorsal view) **11** hypopygium (ventral view) **12** anal point and inferior volsella **13** gonostylus.

*Wing* (Figure 8). VR 1.33–1.43, 1.37. Anal lobe obtuse. Brachiolum with 1 seta; R with 5–13, 9 setae; R_1_ with 1–4, 2 setae; other veins bare. Squama with 1–3, 2 setae. Costal extension 80–84, 81 μm long. Cu_1_ slightly curved.

Thorax (Figure 9). Antepronotum with 4–6, 5 lateral setae, dorsocentrals 9–13, 11, acrostichals 3–7, 5, prealars 3–5, 4. Scutellum with 4–5, 5 setae.

Legs. Pulvilli present. Spur of fore tibia 29–41, 34 μm long, spurs of mid tibia 19–29, 22 μm and 19 μm long; hind tibia with a long spur 36–41, 38 μm long, a short spur 24–31, 27 μm long and comb composed of 10–12, 11 spines. Width at apex of fore tibia 34–46, 37 μm, of mid tibia 29–31, 30 μm, of hind tibia 31–36, 33 μm. Lengths (in μm) and proportions of legs as in Table 3.

**Table 3.** Lengths (in μm) and proportions of legs of *Pseudorthocladius (Pseudorthocladius) cylindratus* sp. n.

	p_1_	p_2_	p_3_
fe	400–450, 423	440–500, 466	430–490, 456
ti	340–380, 360	440–510, 477	520–570, 530
ta_1_	420–460, 437	200–220, 210	310–340, 323
ta_2_	264–280, 271	150–175, 163	156–180, 171
ta_3_	180–190, 183	142–178, 152	140–144, 142
ta_4_	98–101, 100	52–72, 68	67–74, 72
ta_5_	67–72, 69	48–55, 51	45–55, 52
LR	1.11–1.23, 1.19	0.46–0.59, 0.50	0.60–0.62, 0.61
BV	1.64–1.90, 1.86	2.86–3.80, 2.89	2.92–3.04, 2.95
SV	1.74–1.80, 1.76	4.03–4.45, 4.28	3.00–3.05, 3.02
BR	1.83–2.00, 1.92	2.86–2.92, 2.88	3.00–3.57, 3.26

Hypopygium (Figures 10–13). Laterosternite IX with 4–6, 5 setae. Anal point (Figure 12) cylindrical and 45–49, 48 μm long and with 10–13, 11 stout setae, 23–28, 25 μm long. Phallapodeme 31–36, 34 μm long. Transverse sternapodeme 65–67, 66 μm long and convex in the middle. Virga absent. Gonocoxite 117–137, 123 μm long, with 7 setae along inner margin. Inferior volsella (Figure 12) developed and triangular with 3–4, 3 strong marginal setae. Gonostylus (Figure 13) 65–72, 67 μm long, narrowed at base and distal end, widen in the middle. Megaseta 8–10, 9 μm long. HR 1.83–1.90, 1.85. HV 2.74–3.14, 2.78.

Female, pupa and larva unknown.

#### Type materials.

Holotype: ♂ (BDN No.26348), China: Hunan Province, Chenzhou City, Yizhang County, Mang Mountain, 25°24'N, 113°18'E, 22.vii.2004, Chuncai Yan, light trap. Paratypes (5 ♂♂): 4 ♂♂, as holotype; 1 ♂, Hainan Province, Changjiang County, Bawang Mountain, 19°15'36"N, 109°03'18"E, 10.v.1988, Xinhua Wang, sweep net.

#### Etymology.

The specific name is from Latin, *cylindratus*, meaning “in the form of a cylinder”, referring to the cylindrical anal point, which is unique in the genus.

#### Remarks.

The new species resembles *Pseudorthocladius (Pseudorthocladius) amplicaudus* Sæther & Sublette, 1983 in the structure of hypopygium, but the new species can be separated from latter on the basis of main characters in Table 4.

**Table 4.** Main differences between *Pseudorthocladius (Pseudorthocladius) cylindratus* sp. n. and *Pseudorthocladius (Pseudorthocladius) amplicaudus* Sæther & Sublette (1983).

Characters	*Pseudorthocladius (Pseudorthocladius) cylindratus* sp. n.	*Pseudorthocladius (Pseudorthocladius) amplicaudus* Sæther & Sublette
Anal point	45–49 μm long and cylindrical	41 μm long and widen at base
AR	0.65–0.67	1.26
VR	1.33–1.43	1.14
LR_1_	1.11–1.23	0.66

#### Distribution.

The new species is known from Hunan, Hainan Province in Oriental China.

### Pseudorthocladius (Pseudorthocladius) digitus sp. n.

http://zoobank.org/F4701FCB-7F55-481D-8F07-0C64CF7C65BC

http://species-id.net/wiki/Pseudorthocladius_digitus

Figures 14–18

#### Diagnosis.

The male imago can be distinguished from the known species of the genus by the following combination of characters: anal point rounded and reaching beyond the caudal margin of Tergite IX; inferior volsella finger–shaped; squama bare; anal lobe reduced.

#### Description.

Adult male (n = 1). Total length 2.43 mm. Wing length 1.55 mm. Total length/wing length 1.57. Wing length/length of profemur 2.54.

Coloration. Head, abdomen, legs brown; thorax with yellow ground with brown postnotum and preepisternum.

Head. Antenna with 13 flagellomeres. Terminal flagellomere length 300 μm. AR 0.74. Temporal setae 7, including 4 inner verticals, 3 outer verticals. Clypeus with 2 setae. Tentorium 110 μm long, 24 μm wide. Palpomere lengths (in μm): 29, 31, 60, 108, –.

**Figures 14–18.**
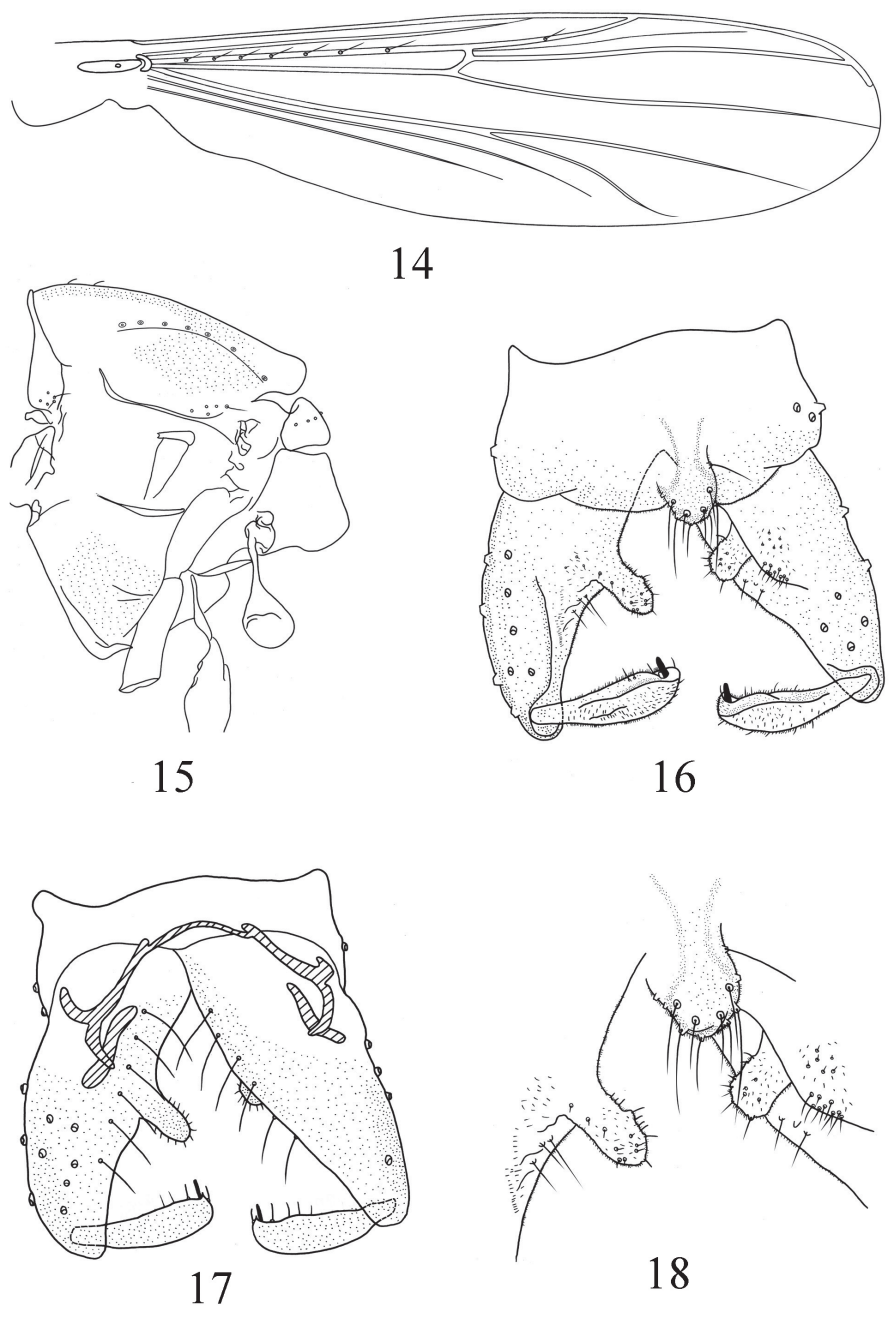
*Pseudorthocladius (Pseudorthocladius) digitus* sp. n., male. **14** wing **15** thorax **16** hypopygium (dorsal view) **17** hypopygium (ventral view) **18** anal point and inferior volsella.

Wing (Figure 14). Anal lobe reduced. Brachiolum with 1 seta; R with 7 setae; R_1_ with 1 seta; other veins bare. Squama bare. Costa extention 41 μm long. Cu_1_ slightly curved.

Thorax (Figure 15). Antepronotum with 5 lateral setae, dorsocentrals 7, acrostichals 2, prealars 5. Scutellum with 9 setae.

Legs. Pulvilli present. Spur of fore tibia 50 μm long, spurs of mid tibia both 29 μm long; hind tibia with a long spur 60 μm long, a short spur 29 μm long and comb composed of 12 spines. Width at apex of fore tibia 43 μm, of mid tibia 36 μm, of hind tibia 45 μm. Lengths (in μm) and proportions of legs as in Table 5.

**Table 5.** Lengths (in μm) and proportions of legs of *Pseudorthocladius (Pseudorthocladius) digitus* sp. n.

	p_1_	p_2_	p_3_
fe	610	660	700
ti	650	660	800
ta_1_	410	264	450
ta_2_	250	146	240
ta_3_	156	101	180
ta_4_	96	67	74
ta_5_	72	60	72
LR	0.63	0.25	0.56
BV	2.91	4.23	3.44
SV	3.07	5.00	3.33
BR	2.63	1.36	2.78

Hypopygium (Figures 16–18). Laterosternite IX with 3 setae. Anal point (Figure 18) rounded and reaching beyond caudal margin of Tergite IX, maximum width 22 μm, with 10 long marginal setae. Phallapodeme 48 μm long. Transverse sternapodeme 50 μm long with small oral projection. Virga absent. Gonocoxite 178 μm long with 6 strong setae along inner margin. Inferior volsella (Figure 18) finger–shaped, parallel–sided and rounded in the apex, bearing some weak setae along the margin and covered by microtrichia. Gonostylus 84 μm long, narrow at base, widen to the distal, with 3–4 setae along inner margin. Crista dorsalis visible, relatively low. Megaseta 10 μm long. HR 2.11. HV 2.89.

Female, pupa and larva unknown.

#### Type materials.

Holotype: ♂ (BDN No.05327), China: Fujian Province, Wuyi City, Wuyi Mountain, 27°45'N, 118°03'E, 26.iv.1993, Xinhua Wang, sweep net.

#### Etymology.

The specific name is from Latin, *digitus*, meaning “finger”, referring to the finger–shaped inferior volsella.

#### Remarks.

*Pseudorthocladius (Pseudorthocladius) digitus* sp. n. is close to *Pseudorthocladius (Pseudorthocladius) yakuxeyeus* (Sasa & Suzuki, 2000) in the antenna ratio (0.71–0.74) and finger–liked inferior volsella. But it can be separated from the latter by having rounded anal point reaching beyond the caudal margin of tergite IX, reduced wing anal lobe and bare squama.

#### Distribution.

The new species is known from Fujian Province in Oriental China.

### Pseudorthocladius (Pseudorthocladius) jintutridecima

(Sasa, 1996)

http://species-id.net/wiki/Pseudorthocladius_jintutridecima

Eukiefferiella jintutridecimus Sasa, 1996: 64.
? Psectrocladius (Mesopsectrocladius) jintutridecima Sæther, Ashe & Murray, 2000: 171.
Pseudorthocladius (Pseudorthocladius) jintutridecima Yamamoto 2004: 82; Ashe and O’Connor 2012: 534.

#### Diagnosis.

AR 0.25–0.96; wing anal lobe near rectangular; tergite IX without anal point, just with a strongly chitinized broad and rounded ridge, bearing 13 strong setae; inferior volsella low and round, posterior corner.

#### Specimens examined.

China, Sichuan: 1 ♂, Baoxing County, 30°24'N, 102°54'E, 19.vi.1996, Ruilei Zhang, light trap. Shaanxi: 1 ♂, Liuba County, 33°39'N, 106°57'E, 1.viii.1994, Bingchun Ji, light trap. Fujian: 2 ♂♂ Wuyi City, Wuyi Mountain, 27°45'N, 118°03'E, 26.iv.1993, Xinhua Wang, light trap. Yunnan: 11 ♂♂, Lijiang City, Lijiang County, Shigu town, Chongjiang river, 26°51'N, 100°14'E, 25.v.1996, Xinhua Wang, light trap; 6 ♂♂, Yulong Naxi Autonomous County, Tiger Leaping Gorge, 27°11'24"N, 100°07'12"E, 26.v.1996, Xinhua Wang, light trap. Guangdong: 1 ♂, Fengkai County, 23°24'N, 111°30'E, 20.iv.1988, Xinhua Wang, sweep net.

#### Remarks.

Sasa (1996) described this species based on the material from Japan, and put it in the genus *Eukiefferiella*. Sæther, Ashe and Murray (2000) transferred this species to the genus ? *Psectrocladius*. Yamamoto (2004) transferred it into the genus *Pseudorthocladius*. The Chinese specimens agree with the original description of Sasa (1996) with exception of Chinese specimens have lower AR (0.25–0.86).

#### Distribution.

Shaanxi, Fujian, Guangdong, Sichuan, Yunnan Province (Oriental China); Japan.

### Pseudorthocladius (Pseudorthocladius) macrovirgatus

Sæther & Sublette, 1983

http://species-id.net/wiki/Pseudorthocladius_macrovirgatus

Pseudorthocladius (Pseudorthocladius) macrovirgatus Sæther & Sublette, 1983: 88; Ashe and O’Connor 2012: 535.
Pseudorthocladius (Pseudorthocladius) cranstoni Sæther & Sublette, 1983: 92; Schnell 1991: 5–10.

#### Diagnosis.

AR 1.04–1.18; R_4+5_ with 0–8 setae, squama with 6–15 setae; virga consisting of 2 broad lateral spines and 4 partially fused median spines, about 0.5×as long as gonostylus; gonostylus well–developed with rounded inferior volsella.

#### Specimens examined.

China, Zhejiang: 1 ♂, Taizhou City, Tiantai County, Baxian Lake, 29°09'N, 120°57'E, 13.iv.2011, Xiaolong Lin, sweep net.

#### Remarks.

The Chinese specimen mainly agrees with the original description by Sæther and Sublette (1983). Some measured differences between the Chinese specimen and the specimen described by Sæther and Sublette (1983) are shown in Table 6.

**Table 6.** Differences between the specimens of China and description of Sæther and Sublette (1983).

	Chinese specimen	Description of Sæther & Sublette
TL	2.58 mm	2.46–2.47 mm
WL	1.50 mm	1.07–1.37 mm
VR	1.08	1.15–1.21
LR_1_	0.62	0.57–0.62
HV	2.68	3.57–3.61

#### Distribution.

Zhejiang Province (Oriental China); Europe (Norway; Great Britain; Ireland; France and Netherlands, and North America (U.S.A. and Canada).

### Pseudorthocladius (Pseudorthocladius) morsei

Sæther & Sublette, 1983

http://species-id.net/wiki/Pseudorthocladius_morsei

Pseudorthocladius (Pseudorthocladius) morsei Sæther & Sublette, 1983: 85; Ashe and O’Connor 2012: 535.

#### Diagnosis.

AR 0.78–0.97; virga consisting of tight cluster of about 10 spines and 2 broader lateral blades; inferior volsella with concave inner margin and 1 anterior and 1posterior corner; gonostylus with basal inner lobe, a sharp bend distad of the middle, and a narrow apical posterior.

#### Specimens examined.

China, Sichuan: 1 ♂, Kangding County, 29°54'N, 102°06'E, 8.vi.1996, Xinhua Wang, light trap.

#### Remarks.

Sæther and Sublette (1983) described *Pseudorthocladius (Pseudorthocladius) morsei* based on the material from U.S.A. Its gonostylus has a basal inner lobe, sharply bend distad of the middle and narrow in apical posterior, which is unique among *Pseudorthocladius*. The Chinese specimen agrees with the description except some minor differences shown in Table 7.

**Table 7.** Differences between the specimen of China and description of Sæther and Sublette (1983).

	Chinese specimen	Description of Sæther & Sublette
TL	2.53 mm	1.97 mm
WL	1.80 mm	1.19 mm
AR	0.97	0.78
VR	1.12	1.09
LR_1_	0.62	0.59
Virga	consisting of tight cluster of about 8 spines and 2 thin lateral spines	consisting of tight cluster of about 10 spines and 2 broader lateral blades
inferior vollsella	rectangular without visible corner	with obvious anterior and posterior corner

#### Distribution.

Sichuan Province (Oriental China); U.S.A.; Canada.

### Pseudorthocladius (Pseudorthocladius) ovatus sp. n.

http://zoobank.org/672C06D7-0A63-4040-BE31-188FCA2FEB77

http://species-id.net/wiki/Pseudorthocladius_ovatus

Figures 19–23

#### Diagnosis.

The male imago can be distinguished from the known species of the genus by the following combination of characters: anal point round baring 9 long and strong setae; inferior volsella oval with round margin and bearing 8 long and stout marginal setae; high AR(1.40).

#### Description.

Adult male (n = 5). Total length 2.90–3.20, 2.98 mm. Wing length 1.43–1.55, 1.47 mm. Total length/wing length 1.88–2.08, 2.00. Wing length/length of profemur 2.26–2.41, 2.35.

Coloration. Head, abdomen, legs brown; thorax with yellow ground with brown preepisternum and brownish black postnotum.

Head. Antenna with 13 flagellomeres. Terminal flagellomere length 410–460, 440 μm. AR 1.31–1.55, 1.40. Temporal setae 8–11, 10, including 3–7, 4 inner verticals, 5–6, 5 outer verticals, and 0–1, 1 postorbital. Clypeus with 8–12, 10 setae. Tentorium 120–132, 126 μm long, 31–33, 32 μm wide. Palpomere lengths (in μm): 31–36, 32; 43–45, 44; 105–108, 107; 155–158, 156; 201–207, 204. L: 5^th^/3^rd^ 1.81–1.96, 1.91.

**Figures 19–23.**
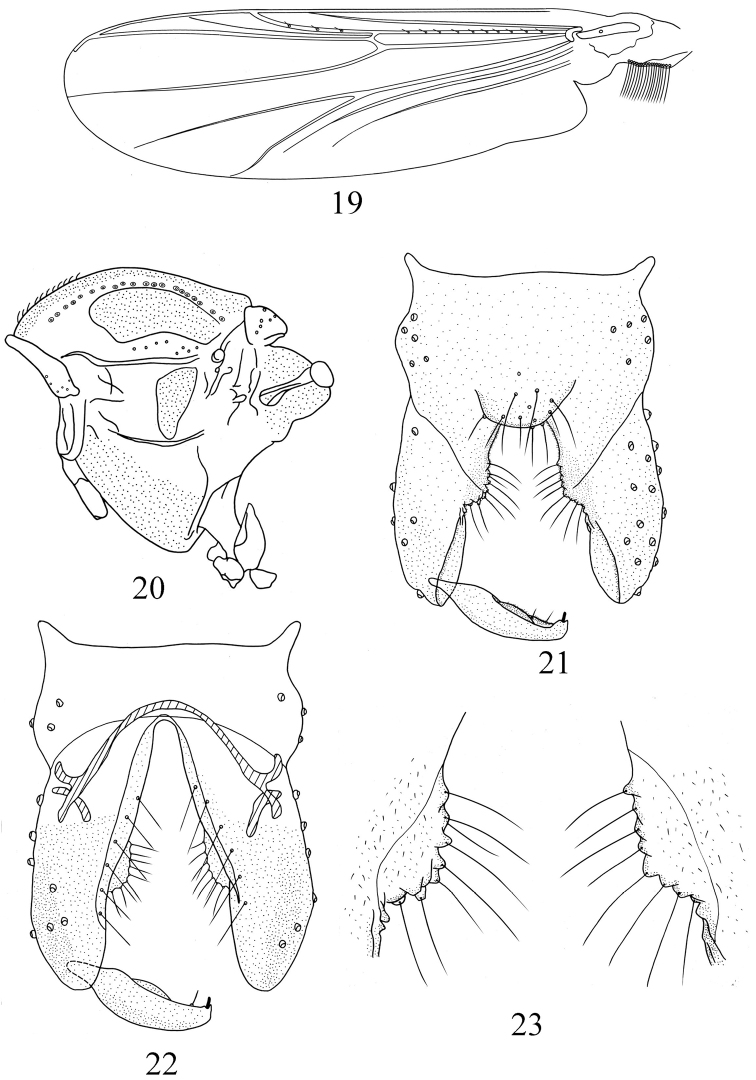
*Pseudorthocladius (Pseudorthocladius) ovatus* sp. n., male. **19** wing **20** thorax **21** hypopygium (dorsal view) **22** hypopygium (ventral view) **23** inferior volsella.

Wing (Figure 19). VR 1.19–1.26, 1.21. Anal lobe well–developed. Brachiolum with 1 seta; R with 10–12, 10 setae; R_1_ with 3–4, 3 setae; other veins bare. Squama 14–18, 16 setae. Costa extension 36–50, 46 μm long. Cu_1_ slightly curved.

Thorax (Figure 20). Antepronotum with 5–8, 6 lateral setae, dorsocentrals 20–25, 22, acrostichals 8–12, 10, prealars 7–8, 8. Scutellum with 12–17, 15 setae.

Legs. Pulvilli present. Spur of fore tibia 45–48, 46 μm long, spurs of mid tibia both 19–24, 22 μm long; hind tibia with a long spur 48–52, 50 μm long, a short spur 17–24, 22 μm long and comb composed of 12–14, 13 spines. Width at apex of fore tibia 38–43, 41 μm, of mid tibia 31–40, 36 μm, of hind tibia 43–48, 45 μm. Lengths (in μm) and proportions of legs as in Table 8.

**Table 8.** Lengths (in μm) and proportions of legs of *Pseudorthocladius (Pseudorthocladius) ovatus* sp. n.

	p_1_	p_2_	p_3_
fe	600–650, 630	640–700, 670	650–700, 678
ti	720–800, 750	620–670, 648	760–850, 825
ta_1_	480–550 (4), 498	250–330, 280	400–500, 562
ta_2_	310–340 (4), 320	122–156, 148	240–260, 250
ta_3_	210–250 (4), 230	100–115, 110	161–210, 192
ta_4_	140–150 (4), 143	60–72, 66	96–110, 102
ta_5_	90–100 (4), 96	60–72, 66	79–90, 87
LR	0.67–0.69 (4), 0.68	0.41–0.46, 0.43	0.53–0.59, 0.56
BV	2.37–2.76 (4), 2.41	4.04–4.25, 4.17	2.70–3.22, 3.11
SV	2.52–2.69 (4), 2.59	4.24–4.37, 4.32	3.08–3.27, 3.19
BR	1.67–2.22 (4), 1.88	2.65–2.78, 2.70	2.27–2.69, 2.54

Hypopygium (Figures 21–23). Laterosternite IX with 6–7, 6 setae. Tergite IX with round anal point, bearing 9–10, 9 long and strong setae. Phallapodeme 36–40, 38 μm long. Transverse sternapodeme 72–84, 81 μm long. Virga absent. Gonocoxite 153–168, 162 μm long with oval inferior volsella (Figure 23) with rounded margin and bearing 8 long, stout marginal setae. Gonostylus 89–96, 98 μm long, with small crista dorsalis. Megaseta 5–7, 6 μm long. HR 1.70–1.89, 1.75. HV 3.18–3.23, 3.22.

Female, pupa and larva unknown.

#### Type materials.

Holotype: ♂ (BDN No.26746), China: Zhejiang Province, Wenzhou City, Taishun County, 27°33'N, 119°39'E, 1.viii.2005, Bingchun Ji, light trap. Paratypes (4 ♂♂): 3 ♂♂, Zhejiang Province, Tianmu Mountain, 30°19'N, 119°26'E, 8.ix.1998, Xinhua Wang, light trap; 1 ♂, Zhejiang Province, Lishui City, Qingyuan county, 27°39'N, 119°09'E, 13.vii.1994, Hong Wu, sweep net.

#### Etymology.

The specific name is from Latin, *ovatus*, meaning egg–shaped, referring to the oval inferior volsella.

#### Remarks.

The new species is close to *Pseudorthocladius (Pseudorthocladius) matusecundus* Sasa & Kawai, 1987 in the structure of hypopygium, but can be separated from the latter on the basis of characters in Table 9.

**Table 9.** Main differences between *Pseudorthocladius (Pseudorthocladius) ovatus* sp. n. and *Pseudorthocladius (Pseudorthocladius) matusecundus* Sasa and Kawai (1987).

	*Pseudorthocladius (Pseudorthocladius) ovatus* sp. n.	*Pseudorthocladius (Pseudorthocladius) matusecundus* Sasa & Kawai
AR	1.31–1.55	1.09
palp segment	5	4
inferior volsella	oval with round margin	small with round margin and a small posterior corner
gonostulus	widest at about basal 1/3	widest at about distal 1/3.

#### Distribution.

The new species is known from Zhejiang Province in Oriental China.

### Pseudorthocladius (Pseudorthocladius) paucus sp. n.

http://zoobank.org/1BEF00CA-4362-49BE-85D9-E161C9E2BDD0

http://species-id.net/wiki/Pseudorthocladius_paucus

Figures 24–30

#### Diagnosis.

The male imago can be distinguished from the known species of the genus by the following combination of characters: with few setae on squama, R_4+5_ and acrostichals; gonostylus expanded at the apex; anal point triangular baring about 7 stout setae; inferior volsella inserted along the gonocoxite, parallel–sided.

#### Description.

Adult male (n = 3). Total length 1.49–1.60 mm. Wing length 0.77–0.92 mm. Total length/wing length 1.74–1.75. Wing length/length of profemur 2.56–2.58.

Coloration. Head, abdomen, legs light brown; thorax with light brown ground with brownish black postnotum and preepisternum.

Head. Antenna with 13 flagellomeres. Terminal flagellomere length 115–153 μm, conspicuous swollen apically and with strong sensory setae. AR 0.26–0.48. Temporal setae 5–9, including 2–4 inner verticals, 3–5 outer verticals. Clypeus with 7–10 setae. Tentorium 72–84 μm long, 14–19 μm wide. Palpomere lengths (in μm): 22–24; 24–26; 48–55; 60–65; 91–96. L: 5^th^/3^rd^ 1.62–1.65.

**Figures 24–30.**
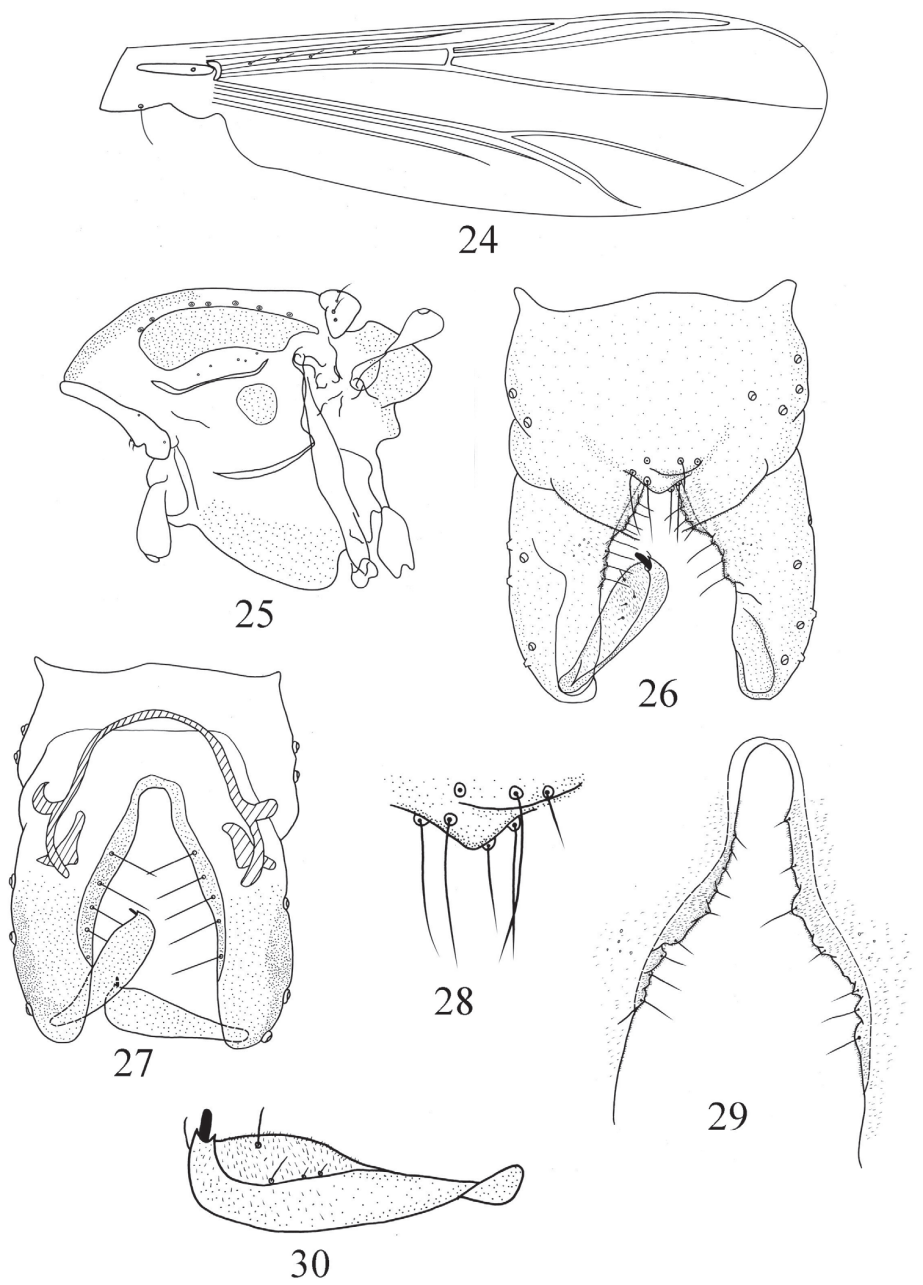
*Pseudorthocladius (Pseudorthocladius) paucus* sp. n., male. **24** wing **25** thorax **26** hypopygium (dorsal view) **27** hypopygium (ventral view) **28** anal point **29** inferior volsella **30** gonostylus.

Wing (Figure 24). VR 1.15–1.27. Anal lobe obtuse. Brachiolum with 1 seta; R with 4 setae; R_4+5_ with 0–1 seta; other veins bare. Squama with 0–1 seta. Costa extension 60 μm long. Cu_1_ slightly curved.

Thorax (Figure 25). Antepronotum with 4–6 lateral setae, dorsocentrals 6–9, acrostichals 0–1, prealars 4–6. Scutellum with 6–7 setae.

Legs. Pulvilli present. Spur of fore tibia 29–34 μm long, spurs of mid tibia both 17–19 μm long; hind tibia with a long spur 31–33 μm long, a short spur 19–24 μm long and comb composed of 10–12, 11 spines. Width at apex of fore tibia 21–26 μm, of mid tibia 21–24 μm, of hind tibia 24–29 μm. Lengths (in μm) and proportions of legs as in Table 10.

**Table 10.** Lengths (in μm) and proportions of legs of *Pseudorthocladius (Pseudorthocladius) paucus* sp. n.

	p_1_	p_2_	p_3_
fe	320–360	320–380	430–490
ti	340–360	350–380	370–410
ta_1_	210–250	140–150	200–230
ta_2_	130–160	72–77	108–120
ta_3_	108–130	55–62	91–96
ta_4_	64–67	36–40	41–46
ta_5_	52–55	41–43	41–48
LR	0.62–0.69	0.35–0.40	0.52–0.56
BV	2.27–2.36	3.88–4.17	3.10–3.54
SV	2.88–3.19	4.79–5.07	3.44–3.65
BR	2.20–2.50	3.20–3.40	3.57–4.00

Hypopygium (Figures 26–30). Laterosternite IX with 3–4 setae. Anal point (Figure 28) subtriangular with round apex, 9–10 μm long and 24–26 μm wide, with 4–5 lateral setae and 2–5 long setae around the base. Phallapodeme 22–24 μm long. Transverse sternapodeme 43–48 μm long. Virga absent. Gonocoxite 77–89 μm long with reduced parallel–sided inferior volsella (Figure 29). Gonostylus (Figure 30) 43–50 μm long, expanded at the apex, crista dorsalis reduced. Megaseta 5 μm long. HR 1.60–1.83. HV 2.90–3.25.

Female, pupa and larva unknown.

#### Type materials.

Holotype: ♂ (BDN No.25207), China, Hunan, Hengyang County, Heng Mountain, 27°15'N, 112°51'E, 19.vii.2004, Chuncai Yan, sweep net. Paratypes: 2 ♂♂, Hunan, Dong’an Couny, Shunhuang Mountai, 26°24'N, 111°18'E, 26.vii.2004, Chuncai Yan, sweep net.

#### Etymology.

The specific name is from Latin, *paucus*, meaning “few”, referring to the new species has few setae on squama, R_4+5_ and acrostichals.

#### Remarks.

The new species resembles *Pseudorthocladius (Pseudorthocladius) oyabecrassus* Sasa, Kawai & Ueno, 1988 in the low AR (0.43, 0.50), gonostylus strongly expanded at about distal, but can be separated from *Pseudorthocladius (Pseudorthocladius) oyabecrassus* on the basis of characters in table 11.

#### Distribution.

The new species is known from Hunan Province in Oriental China.

**Table 11.** Main different characters between *Pseudorthocladius (Pseudorthocladius) paucus* sp. n. and *Pseudorthocladius (Pseudorthocladius) oyabecrassus* Sasa, Kawai & Ueno (1988).

	*Pseudorthocladius (Pseudorthocladius) paucus* sp. n.	*Pseudorthocladius (Pseudorthocladius) oyabecrassus* Sasa, Kawai & Ueno
Body length	1.49–1.60 mm	2.18 mm
Acrostichals	0–1	11
Anal point	subtriangular with round apex	conical, darkly pigment
Pulvilli	present	absent
Inferior volsella	parallel–sided	with a small projection

### Pseudorthocladius (Pseudorthocladius) uniserratus

Sæther & Sublette, 1983

http://species-id.net/wiki/Pseudorthocladius_uniserratus

Pseudorthocladius (Pseudorthocladius) uniserratus Sæther & Sublette, 1983: 71; Ashe and O’Connor 2012: 538.

#### Diagnosis.

AR 0.63–0.95; R with 3–13 setae, R_1_ with 0–6 setae, R_4+5_ with 0–13 setae; squama with 4–6 setae; inferior volsella trianguler at middle; virga consisting of very weak field of about 20 minute spinules; crista dorsalis low, HR 1.68–1.91, HV 2.53.

#### Specimens examined.

China: Hunan Province: 1 ♂, Chenzhou City, Yizhang County, Mang Mountain, 25°24'N, 113°18'E, 22.vii.2004, Chuncai Yan, sweep net.

#### Remarks.

Sæther and Sublette (1983) described the male imago, pupa and larva from the U.S.A. The Chinese specimens mainly agree with the adult description of Sæther and Sublette (1983). According to Sæther and Sublette (1983), there are not significant differences between *Pseudorthocladius (Pseudorthocladius) uniserratus* and *Pseudorthocladius (Pseudorthocladius) curtistylus* in the male hypopygium, but as we can see in the figures of *Pseudorthocladius (Pseudorthocladius) uniserratus*, the gonostylus is expanded at about distal while *Pseudorthocladius (Pseudorthocladius) curtistylus* is narrowed; the inferior volsella is triangler at middle part. The Chinese specimens agree with *Pseudorthocladius (Pseudorthocladius) uniserratus* more.

#### Distribution.

Oriental China (Hunan Province); U.S.A.; Canada.

### Pseudorthocladius (Lordella) wingoi

Sæther & Sublette, 1983

http://species-id.net/wiki/Pseudorthocladius_wingoi

Pseudorthocladius (Lordella) wingoi Sæther & Sublette, 1983: 98; Ashe and O’Connor 2012: 530.
Pseudorthocladius (Lordella) comans Sæther & Sublette, 1983: 95; Cranston and Oliver 1988: 446.

#### Diagnosis.

Inferior volsella hook-liked, bended posteriad; gonostylus broadest at base and densely covered with microtrichia; virga with 5–8 stronger spines and 0–20 finer spinules; AR 0.8–1.1; dorsocentrals 10–16; R with 1–3 setae, exceptionally with 12 setae.

#### Specimens examined.

China, Guizhou: 8 ♂♂, Fanjing Mountain, 27°54'54"N, 108°41'42"E, 28.v.–3.vi.2002, Bingchun Ji, sweep net.

#### Remarks.

The Chinese specimens mainly agree with the description by Sæther and Sublette (1983). According to Cranston and Oliver (1988), *Pseudorthocladius (Lordella) comans* is a synonym of *Pseudorthocladius (Lordella) wingoi*. Based on the specimens from Canada the shape of the gonostylus is highly dependent on orientation and the spines might be correlated with size, so *Pseudorthocladius (Lordella) comans* and *Pseudorthocladius (Lordella) wingoi* should be the same species. The minor differences between Chinese specimens and North America specimens are as follows: (1) The anal point is a little shorter (12–17 μm); (2) with small AR (0.75–0.86); (3) dorsocentrals 7–8.

#### Distribution.

Oriental China (Guizhou Province); U.S.A.; Canada.

### Key to males of the genus *Pseudorthocladius* from China

**Table:** 

1	Inferior volsella hook–liked, curved posteriad; gonostylus broadest at base and densely covered with microtrichia	*Pseudorthocladius (Lordella) wingoi* Sæther & Sublette
–	Inferior volsella rounded, or bluntly triangular, never hook-shaped; gonostylus widest some distance from base or at apex, with less conspicuous microtrichia	*Pseudorthocladius* subg. *Pseudorthocladius*
2	Wing membrane densely covered with setae	*Pseudorthocladius (Pseudorthocladius) cristagus* Stur & Sæther
–	Wing membrane bare	3
3	Gonostylus with median sharp bend and tapering apex	*Pseudorthocladius (Pseudorthocladius) morsei* Sæther & Sublette
–	Gonostylus without median bend and tapering apex	4
4	Inferior volsella finger-shaped	5
–	Inferior volsella rounded or triangular, never finger–shaped	6
5	Squama with 8 setae, inferior volsella with about 6 stout setae, transverse sternapodeme without oral projection	*Pseudorthocladius (Pseudorthocladius) jintutridecima* (Sasa)
–	Squama bare, inferior volsella with few weak setae, transverse sternapodeme with oral projection	*Pseudorthocladius (Pseudorthocladius) digitus* sp. n.
6	Virga consisting of 2 broader lateral spines and 4 partially fused median spines	*Pseudorthocladius (Pseudorthocladius) macrovirgatus* Sæther & Sublette
–	Virga absent, if present then consisting of fine spinules	7
7	Anal point long and cylindrical	*Pseudorthocladius (Pseudorthocladius) cylindratus* sp. n.
–	Anal point round or subtriangular	8
8	Inferior volsella with two lobes	*Pseudorthocladius (Pseudorthocladius) binarius* sp. n.
–	Inferior volsella with only one lobe	9
9	AR 0.26–0.48, acrostichal 0–1, R with 4 setae, dorsocentrals 6–9	*Pseudorthocladius (Pseudorthocladius) paucus* sp. n.
–	AR ≤ 0.64 or ≥ 1.31, acrostichals 8–14, R with 10–13 setae, dorsocentrals 12–25	10
10	AR 1.31–1.55, squama 14–18, dorsocentrals 20–25, anal point rounded	*Pseudorthocladius (Pseudorthocladius) ovatus* sp. n.
–	AR 0.45–0.64, squama 3–4, dorsocentrals 12–18, anal point triangular	11
11	AR 0.45–0.51, crista dorsalis rounded and protruding	*Pseudorthocladius (Pseudorthocladius) curtistylus* (Goetghebuer)
–	AR 0.63–0.81, crista dorsalis lower and less rounded	*Pseudorthocladius (Pseudorthocladius) uniserratus* Sæther & Sublette

## Supplementary Material

XML Treatment for Pseudorthocladius (Pseudorthocladius) binarius

XML Treatment for Pseudorthocladius (Pseudorthocladius) cristagus

XML Treatment for Pseudorthocladius (Pseudorthocladius) curtistylus

XML Treatment for Pseudorthocladius (Pseudorthocladius) cylindratus

XML Treatment for Pseudorthocladius (Pseudorthocladius) digitus

XML Treatment for Pseudorthocladius (Pseudorthocladius) jintutridecima

XML Treatment for Pseudorthocladius (Pseudorthocladius) macrovirgatus

XML Treatment for Pseudorthocladius (Pseudorthocladius) morsei

XML Treatment for Pseudorthocladius (Pseudorthocladius) ovatus

XML Treatment for Pseudorthocladius (Pseudorthocladius) paucus

XML Treatment for Pseudorthocladius (Pseudorthocladius) uniserratus

XML Treatment for Pseudorthocladius (Lordella) wingoi
